# Serum 25-Hydroxyvitamin D Deficiency in Chinese Patients with Vitiligo: A Case-Control Study

**DOI:** 10.1371/journal.pone.0052778

**Published:** 2012-12-27

**Authors:** Xin Xu, Wen-Wen Fu, Wen-Yu Wu

**Affiliations:** Department of Dermatology, Huashan Hospital, Fudan University, Shanghai, China; Statens Serum Institute, Denmark

## Abstract

**Background:**

Low vitamin D levels have been noted in patients with a variety of autoimmune diseases. A recent study showed that low vitamin D levels may be associated with vitiligo.

**Objectives:**

To assess 25-hydroxyvitamin D (25(OH)D) status in Chinese patients with vitiligo in comparison of normal controls and explore possible affecting factors.

**Methods:**

We performed a case-control study including 171 patients, 50 controls in 25(OH)D lowest months and 30 patients, 20 controls in 25(OH)D highest months. Demographic and clinical variables of patients were analyzed to determine the correlation with 25(OH) D levels.

**Results:**

25(OH)D mean value of patients was highest in September and October, lowest in March. None of the patients and normal controls had ‘sufficient’ 25(OH)D (> = 75 nmol/L). No significant difference was found in either 25(OH)D mean values or insufficiency/deficiency ratio between patients and controls in 25(OH)D highest and lowest periods. Female patients were at a higher risk of 25(OH)D deficiency than male patients(P = 0.019). For non-segmental type, patients with 25(OH)D deficiency were more likely to have autoimmune thyroid disease than those with insufficiency (P = 0.016). Sex (P = 0.035), thyroid conditions (p = 0.034), testing month (p = 0.049) were independent factors affecting 25(OH)D level in multivariate analysis.

**Conclusion:**

Chinese population lack 25(OH)D universally. 25(OH)D level shows no correlation with onset of vitiligo in Chinese. However deficient 25(OH)D level may be linked to autoimmune disorders in patients.

## Introduction

Recently vitamin D has been found immune-protective. It inhibits the maturation of DCs, regulates related cytokines to shift Th1 response to Th2 response. It inhibits Th17 cells, increases Treg cells to suppress auto-attack and maintain self-tolerance [1]. Reduced vitamin D levels have been associated with many autoimmune diseases. Adequate supplementation may improve the prognosis [2].

Vitiligo is considered to be autoimmunity related. Vitamin D analogues are effective topical therapies for cutaneous autoimmune conditions including psoriasis and vitiligo. In 2010 Silverberg et al assessed serum 25-hydroxyvitamin D (25(OH)D) levels in 45 patients with vitiligo [3]. 55.6% were insufficient(22.5–75 nmol/L), 13.3% were very low (<.22.5 nmo/L). Darker-skinned patients and patients with comorbid autoimmune diseases were more likely to have very low 25(OH)D levels, indicating low vitamin D levels may be associated with vitiligo.

Chinese population are mainly Fitzpatrick phototype III and IV with an increased risk of vitamin D insufficiency. Vitamin D supplementation may help the patients. With this background we conducted a case-control study to compare serum vitamin D levels in Chinese patients with normal people and analyzed the possible affecting factors. As a result, we demonstrated the vitamin D state of Chinese patients compared with normals and affecting factors.

## Materials and Methods

### Ethics Statement

The study was approved by the ethic committee of Huashan Hospital affiliated to Fudan University. All participants provided their written informed consent to participate in the study.

### Subjects recruitment

All the patients with vitiligo were randomly gathered from dermatology clinic of Huashan Hospital affiliated to Fudan University. Controls were recruited from healthy volunteers. As vitamin D has an obvious relation with months, we included 171 patients, 50 controls and 30 patients, 20 controls respectively in case-control study in vitamin D lowest and highest months which were also defined in this study (from March to May 2011, from Sep to Oct 2010). In other months from Sep 2010 to Aug 2011(except Feb), we collected another 79 patients. The total 280 patients were used for analysis of possible affecting factors.

Inclusion criteria for patients were: 1) 18–60 yrs, 2) no major cardiovascular, liver, kidney or digestive disease, 3) no treatment for vitiligo one month before testing, 4) no oral vitamin D supplementation one month before testing. Inclusion criteria for controls were the same except for the absence of vitiligo and other autoimmune diseases.

### Methods

Patients information was collected by one dermatologist, including sex, age, type of vitiligo, new/old onset, stage, body surface area affected, comorbid autoimmune diseases, family history of vitiligo and testing month.

Patients collected from Mar to May were randomly tested for thyroid hormones (TSH, FT3, FT4) and antibodies (TPO, ATG).

Patients' blood samples were tested for 25(OH)D levels in the nuclear department of Huashan Hospital at the same week when it was collected.

According to the standard recommended worldwide, we divided 25(OH)D levels into ‘sufficiency’(> = 75 nmol/L), ‘insufficiency’(> = 25 nmol/L but <75 nmol/L) and ‘deficiency’(<25 nmol/L).

In order to avoid 25(OH)D variation by month, the patients and controls in case-control study were all collected in the two specified periods of the year. To determine if the possible difference of 25(OH)D levels between patients and controls varies by month, we analyzed data of the both two periods. Otherwise in case-control study we all used data from Mar to May. Patients and controls in univariate analysis for sex, age, type of vitiligo, stage, comorbid autoimmune disease were from Mar to May, as we collected the most cases in this period. In univariate analysis for testing month and multivariate analysis, all 280 patients collected were involved.

### Statistical analysis

All the data were processed by SPSS 16.0 Software (SPSS Inc, Chicago). T test and Chi-square test were used in comparison between patients and controls and univariate analysis for sex, age, type, stage, comorbid autoimmune diseases. Anova was used in univariate analysis for testing month. Bilateral regression models were used in multivariate analysis. Two-sided P value less than 0.05 was taken to indicate statistical significance for all estimates.

## Results

The study assessed 280 patients, including 135 males and 145 females, 18–60 years of age (mean = 35.6years).

**Figure 1:**
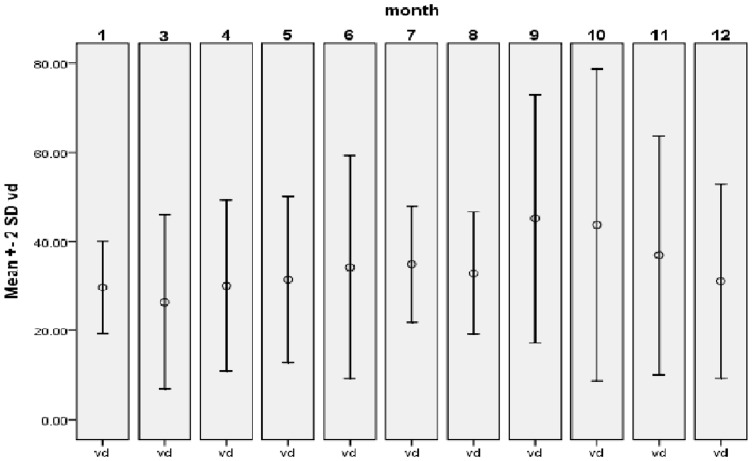
Mean 25(OH)D value of each month through the year (except Feb). The lowest was in March, 26.1±9.7 nmol/L, highest was in September and October, 45.1±14.0 nmol/L-44.1±15.2 nmol/L.

25(OH)D levels were associated with testing month. We accessed 280 patients throughout the year (except Feb) to get 25(OH)D mean value for each month (Figure 1). Data showed 25(OH)D level was highest in September and October which was 45.1±14.0 nmol/L-44.1±15.2 nmol/L, then went down to the lowest in March which was 26.1±9.7 nmol/L.

Characteristics of the 171 patients recruited from Mar to May 2011 are listed in Table 1. Among them, 151 patients were randomly tested for thyroid hormones and antibodies. 12 of the 151 patients were segmental type, but none of them was involved in abnormal test results. So we excluded segmental type when calculating percentage of patients with autoimmune thyroid disease. Finally 28.1% of patients had Hashimoto thyroiditis. No other comorbid autoimmune disease was found.

**Table 1:** Characteristics of the 171 patients included in case-control study from March to May.

Characteristics	Number
Sex	
Male	80
Female	91
Age(yrs,mean = 34.1 yrs)	
18–29	65
30–44	72
45–60	34
Onset	
New	43
Old	128
Type	
Segmental	13
Focal	47
Generalized	102
Universal	2
Halo nevi	7
Stage	
Progressive	120
Stable	51
Surface area affected	
<5%	152
5%–19%	17
> = 20%	2
Comorbid autoimmune diseases (among the 151 patients tested for thyroid hormones and antibodies)	
Hashimoto thyroiditis	39
Family history	5

In control group from Mar to May, 21 were male, 29 were female. 18–60 years of age (the mean = 34.6). There was no significant difference between sex and age of the two groups.

Characteristics of the 30 patients and 20 controls in Sep and Oct are not listed here. No significant difference between sex and age of the two groups.

From Mar to May, 25(OH)D levels of the patients group spread from 8.7 to 63.8 nmol/L, the mean was 29.9±9.6 nmol/L. 114 patients were insufficient (66.9%), 57 were deficient (33.1%). Among the controls, 25(OH)D levels spread from 16.7 to 53.6 nmol/L, the mean was 30.5±8.6 nmol/L. 36 controls were insufficient (72%), 57 were deficient (28%). Patients had a slightly lower mean value of 25(OH)D, a slightly higher percentage of deficiency, but no significant difference was found.

From Sep to Oct, 25(OH)D levels of the patients group spread from 18.8 to 68.8 nmol/L, the mean was 45.1±14.6 nmol/L. 27 patients were insufficient (90%), 3 were deficient (10%). Among the controls, 25(OH)D levels spread from 20.5 to 74 nmol/L, the mean was 48±8.6 nmol/L. 18 controls were insufficient (90%), 2 were deficient (10%). No significant difference was found.

**Figure 2:**
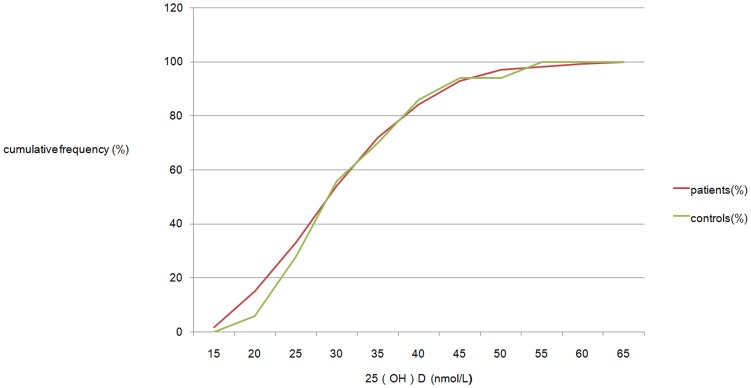
Distribution pattern of 25(OH)D levels in patients and controls from March to May 2011. The horizontal axis represents 25(OH)D levels (nmol/L), the vertical axis represents cumulative frequency of 25(OH)D levels (%). Starting from ‘15 nmol/L’, the largest gap is on ‘20 nmol/L’ point (patients 15.1%, controls 9%, P>0.05).

Looking at the distribution pattern of 25(OH)D levels of the two groups from Mar to May (Figure 2), two line overlapped, the relatively widest gap was in ‘<20 nmol/L’ area, meaning if we use 20 nmol/L as a cut-off point instead of 25 nmol/L, the difference would be the largest, but still not significant.

In both groups from Mar to May, mean 25(OH)D levels of males were slightly above that of females with no significant difference (in patient group, males: 31.8±9.7 nmol/L, females: 28.6±9.3 nmol/L. In control group, males: 33.8±10.7 nmol/L, females: 28.1±5.8 nmol/L). If measured by insufficiency or deficiency, 23.8% of males and 40.7% of females in patient group were deficient while 28.5% of males and 27.6% of females were deficient in control group. Females were at an increased risk of 25(OH)D deficiency than males among patients (P = 0.019).

As mentioned earlier, we did not find comorbid autoimmune disease in segmental type. So we included only non-segmental type in terms of the relationship of 25(OH)D level to comorbid autoimmune diseases. From Mar to May, 37.8% of patients with 25(OH)D deficiency had Hashimoto thyroiditis. 15.6% among them had developed to hypothyroidism (elevated TSH level or lower FT3, FT4). 22.3% of patients with 25(OH)D insufficiency had Hashimoto thyroiditis. 3.2% among them had developed to hypothyroidism. For non-segmental type, patients with 25(OH)D deficiency were at a higher risk of Hashimoto thyroiditis, especially hypothyroidism as a result of thyroiditis (P = 0.016). If non-segmental patients were subdivided into focal, generalized, universal and halo nevi type, the proportion with Hashimoto thyroiditis were 26/94, 8/38, 2/2, 3/5. It seems that patients of universal and halo nevi type had a higher proportion of thyroiditis, but as sample sizes of these two types were too small, statistical conclusions can't be drawn.

We divided patients and controls into three age groups, 18–29 yrs, 30–44 yrs and 45–60 yrs. 25(OH)D mean value of the three age groups were 26.3 nmol/L, 32.2 nmol/L, 34.2 nmol/L in controls and 29.6 nmol/L, 30.6 nmol/L, 30.4 nmol/L in patients. No difference was found between age groups within patients and controls respectively. And no significant difference was found between age-matched groups in patients and controls.

No significant difference was found between segmental/non-segmental type, progressive/stable stage respectively. As universal type and halo nevi had too small sample size, we can't compare between subtypes (segmental type: 29.6 nmol/L, non-segmental type: 30.2 nmol/L, focal type: 29.8 nmol/L, generalized type: 30.0 mol/L, universal type: 32.5 nmol/L, halo nevi: 30.6 nmol/L. Deficiency rate was 23.1%, 34.2%, 37.3%, 31.9%, 0/2, 1/7 in sequence. progressive stage: 30.8 nmol/L, stable stage: 29.5 nmol/L).

Multivariate analysis were performed to examine relationship of 25(OH)D level to all the independent variables in which the independent variables were simultaneously adjusted for each other. By using 25 nmol/L as a cut-off point, multivariate study showed an association of patients' 25(OH)D levels with testing month(P = 0.049), sex(P = 0.035) and thyroid condition(P = 0.034). The three variables can independently affect risk of 25(OH)D deficiency in patients.

## Discussion

Up to now there is no other case-control study about serum vitamin D levels of patients with vitiligo. In previous case-control studies about the association between vitamin D levels and other autoimmune diseases including systemic lupus erythematosus, psoriasis, inflammatory bowel disease, cutaneous lupus erythematosus, rheumatoid arthritis [4]–[8], significant differences were all found in vitamin D level between patients and controls except for rheumatoid arthritis. But these studies were all aimed at light-skinned people and Europeans, no East-Asian people were involved. A latest case-control study aimed at African American patients with cutaneous lupus erythematosus showed no significant difference in vitamin D levels between them and normal African Americans [9]. That is similar to our results. The above article indicated ethnicity may play a greater role in affecting vitamin D levels than CLE status. In our study, we involved a large number of subjects from both vitamin D highest and lowest months, to clearly demonstrate that there was no difference in vitamin D levels between vitiligo patients and controls throughout the year. Our data do not reveal a correlation between vitamin D levels and onset of vitiligo, therefore do not support a role for vitamin D in vitiligo pathogenesis. But more studies are needed to determine if ethnicity matters in the case.

The effect vitamin D has on autoimmunity has been confirmed by many researches. However, when East-Asians are more easily to lack vitamin D than light-skinned people and Europeans, the incidence of autoimmune diseases is not higher. Compared with Silverberg's study about American patients with a lot of races [3], patients in our study showed an increased percentage of low vitamin D levels (100% versus 67.8%), but no increased percentage of comorbid autoimmune diseases (28.1% versus 33.3%). It seems low vitamin D levels of Chinese patients may not have a link with autoimmunity. Due to the fact that vitamin D deficiency is associated with autoimmune thyroid diseases, we suppose for Chinese patient, insufficient-but-not-deficient vitamin D level is not associated with autoimmunity.

On the other hand, our study demonstrated that patients with vitamin D deficiency were at an increased risk of autoimmune thyroid disease than those with insufficiency, which is consistent with Silverberg's study, indicating the association between vitamin D deficiency and autoimmunity. So for patients with deficient vitamin D levels, autoimmune conditions should be evaluated.

Females are associated with lower vitamin D levels, which may be related to the effect of estrogen and less sun exposure. It has been suggested that vitamin D and estrogen levels influence each other and collaborate to maintain Ca homeostasis [10]. Correction of estrogen deficiency in postmenopausal women has been associated with increased 1,25(OH)_2_D production [11]. In our study, we didn't find difference between postmenopausal women and younger women. It may be explained by limited sun exposure among younger women, which cover up the possible difference by contrast with postmenopausal women.

We get the data of 25(OH)D mean values of patients in each month through the year. It demonstrated a close relation with seasons. But even in the peak month no patients can reach the standard of ‘sufficiency’. As vitamin D level itself fluctuates monthly, when setting ‘deficient’ 25(OH)D level to evaluate if extra attention should be paid to autoimmunity, the data could be referred to. But still more samples are needed to determine a precise level of vitamin D for each month.

In conclusion, Chinese population lack vitamin D universally. 25(OH)D shows no correlation with onset of vitiligo in Chinese. 25(OH)D insufficiency is probably not linked to autoimmunity for Chinese patients, however, 25(OH)D deficiency may be associated with autoimmune disorders.
